# A field rice panicle detection model based on improved YOLOv11x

**DOI:** 10.3389/fpls.2025.1656505

**Published:** 2025-09-02

**Authors:** Yuzhu Luo, Xinyu Li, Bing Bai, Xiaoyu Yu, Yu Wang, Zuobin Ma, Liying Zhang, Xiuyuan Peng

**Affiliations:** ^1^ Institute of Information, Liaoning Academy of Agricultural Sciences, Shenyang, China; ^2^ Liaoning Rice Research Institute, Shenyang, China

**Keywords:** field rice, panicle detection, YOLOv11x, UAV image, SK attention, feature fusion

## Abstract

Rice serves as the staple food for over 50% of the world's population, making its yield prediction crucial for food security. The number of panicles per unit area is a core parameter for estimating rice yield. However, traditional manual counting methods suffer from low efficiency and significant subjective bias, while unmanned aerial vehicle (UAV) images used for panicle detection face challenges such as densely distributed panicles, large scale variations, and severe occlusion. To address the above challenges, this paper proposes a rice panicle detection model based on an improved You Only Look Once version 11x (YOLOv11x) architecture. The main improvements include: 1) Introducing a Bi-level Routing Attention (BRA) mechanism into the backbone network to improve the feature representation capability for small objects; 2) Adopting a Transformer-based detection head (TransHead) to capture long-term spatial dependencies; 3) Integrating a Selective Kernel (SK) Attention module to achieve dynamic multi-scale feature fusion; 4) Designing a multi-level feature fusion architecture to enhance multi-scale adaptability. Experimental results demonstrate that the improved model achieves an mAP@0.5 of 89.4% on our self-built dataset, representing a 3% improvement over the baseline YOLOv11x model. It also achieves a Precision of 87.3% and an F1-score of 84.1%, significantly outperforming mainstream algorithms such as YOLOv8 and Faster R-CNN. Additionally, panicle counting tests conducted on 300 rice panicle images show that the improved model achieves R^2^ = 0.85, RMSE = 2.33, and rRMSE = 0.13, indicating a good fitting effect. The proposed model provides a reliable solution for intelligent in-field rice panicle detection using UAV images and holds significant importance for precise rice yield estimation.

## 1 Introduction

As a staple food for over 50% of the world’s population, rice plays a crucial role in global food security (Mohidem et al., 2022). In the 2023/24 crop year, the world’s rice production amounted to roughly 523.8 million tons, with a cultivated area of about 168 million hectares. Fluctuations in rice yield directly influence the global food security landscape. Rice yield has three key components: panicle number, grain number per panicle, and grain weight. Among these, the number of panicles per unit area is the core phenotypic parameter most easily regulated by field management (Slafer et al., 2014; Ferrante et al., 2017). Traditional manual methods for counting panicles face bottlenecks such as being time-consuming, labor-intensive, subjective, and lacking standardization. These limitations make it challenging to fulfill the requirements of high-throughput phenotypic analysis in smart agriculture. Therefore, developing automated, high-precision rice panicle detection technology has become a critical entry point for breaking through the limitations of yield prediction accuracy and achieving precision agricultural management.

Image processing and machine learning are currently the primary technical approaches for panicle detection (Lin and Guo, 2020; Chen et al., 2023; Santiago et al., 2024). By extracting features such as color, shape, and texture from RGB images of rice panicles, machine learning methods can be used to establish panicle detection models for identifying and counting panicles. Duan et al. (2015) proposed a new method for detecting rice panicles at the heading stage through multi-angle color image analysis of rice plants. Their workflow included extracting the i2 plane from original color images, segmenting the images, identifying panicles using artificial neural networks, and calculating the number of panicles in the current image. Wu et al. (2019) developed an image analysis method based on a linear regression model to estimate the number of grains per rice panicle, achieving accuracy rates of 96% and 97% for japonica and indica rice, respectively. Carlier et al. (2022) combined RGB and multispectral image features of wheat ears and assessed three algorithms including random forest, multi-layer perceptron, and support vector machine based on superpixel classification, which realized automatic segmentation of wheat ears at the pixel level. Although machine learning based panicle counting methods possess advantages such as high speed, high throughput and nondestructive detection (Tan et al., 2023), they require manual feature extraction and demonstrate inadequate robustness against noise like uneven illumination and complex backgrounds in field conditions. These limitations reduce the algorithms’ generalization ability and hinder their wider application.

In recent years, the rapid development of object detection algorithms based on deep learning has provided new solutions for rice panicle detection (Xiao et al., 2020; Xu et al., 2020; Deng et al., 2021). Zhang et al. (2021) improved Faster R-CNN by incorporating multiple optimization strategies including dilated convolution, K-means clustering and ROIAlign, proposing an improved Faster R-CNN based panicle detection method. This method achieved an mAP of 80.3%, representing a 2.4% improvement over the original model. Sun et al. (2022) developed a general detection model for curved panicles based on YOLOv4, enabling UAV image recognition of hybrid indica rice in complex field backgrounds. Lu et al. (2024) established an automatic plot segmentation algorithm and constructed a Panicle-ViT instance segmentation network combining ViT with Mask R-CNN, developing an automated, high-throughput method for field plot segmentation and panicle quantification. Wang et al. (2022) proposed a panicle detection and counting method based on deep learning for large-scale field images that effectively preserves small panicle features. Liang et al. (2024) enhanced YOLOv5 by incorporating two attention mechanisms including shuffle attention and gather excite attention, and replacing standard convolutions with GSConv, ultimately developing a rotated panicle detection model for field rice yield estimation. Seng et al. (2025) replaced the original C3 module in YOLOv5 with VoVGSCSP and substituted the backbone with lightweight GhostNet to reduce computational complexity, proposing the YOLOv5s-Slim Neck-GhostNet panicle recognition model. This optimized model achieved an mAP of 97.2% on test sets, showing 1.8% improvement over baseline while reducing model size by 5.7M parameters.

Unmanned aerial vehicles (UAVs), characterized by their compact size, lightweight design, simple operation, low maintenance costs, and high flexibility (Shao et al., 2021), have emerged as a novel lowaltitude remote sensing platform. Equipped with high-resolution RGB cameras, multispectral cameras, or LiDAR sensors, UAVs are capable of swiftly collecting massive amounts of the necessary image data, making them an important tool for agricultural data collection. Consequently, UAVs have seen widespread application in agricultural fields in recent years (Cai et al., 2022; Lin et al., 2025; Song et al., 2025). While UAVs significantly enhance the scope and efficiency of data acquisition, they also present certain limitations in image quality. Additionally, low-altitude aerial images of rice fields exhibits several challenging characteristics: dense panicle distribution, a high proportion of small objects, and severe occlusion between plants. These factors pose new challenges to the accuracy of panicle detection models in UAV-based scenarios (Zhao et al., 2021).

As of now, YOLOv11 is the latest version in the YOLO series, integrating improved model architecture designs, enhanced feature extraction techniques, and optimized training methodologies based on previous iterations. Through these refinements, YOLOv11 delivers superior feature extraction capabilities, faster processing speeds, and higher accuracy with reduced parameter requirements. Therefore, this paper takes YOLOv11x as the fundamental network, improves the backbone, detection head, and feature fusion parts, and incorporates SK Attention to establish a field rice panicle detection model based on the improved YOLOv11x. The aim is to enhance the accuracy of rice panicle detection in UAV images of field rice.

## 2 Materials and methods

### 2.1 UAV image acquisition

**Figure 1:**
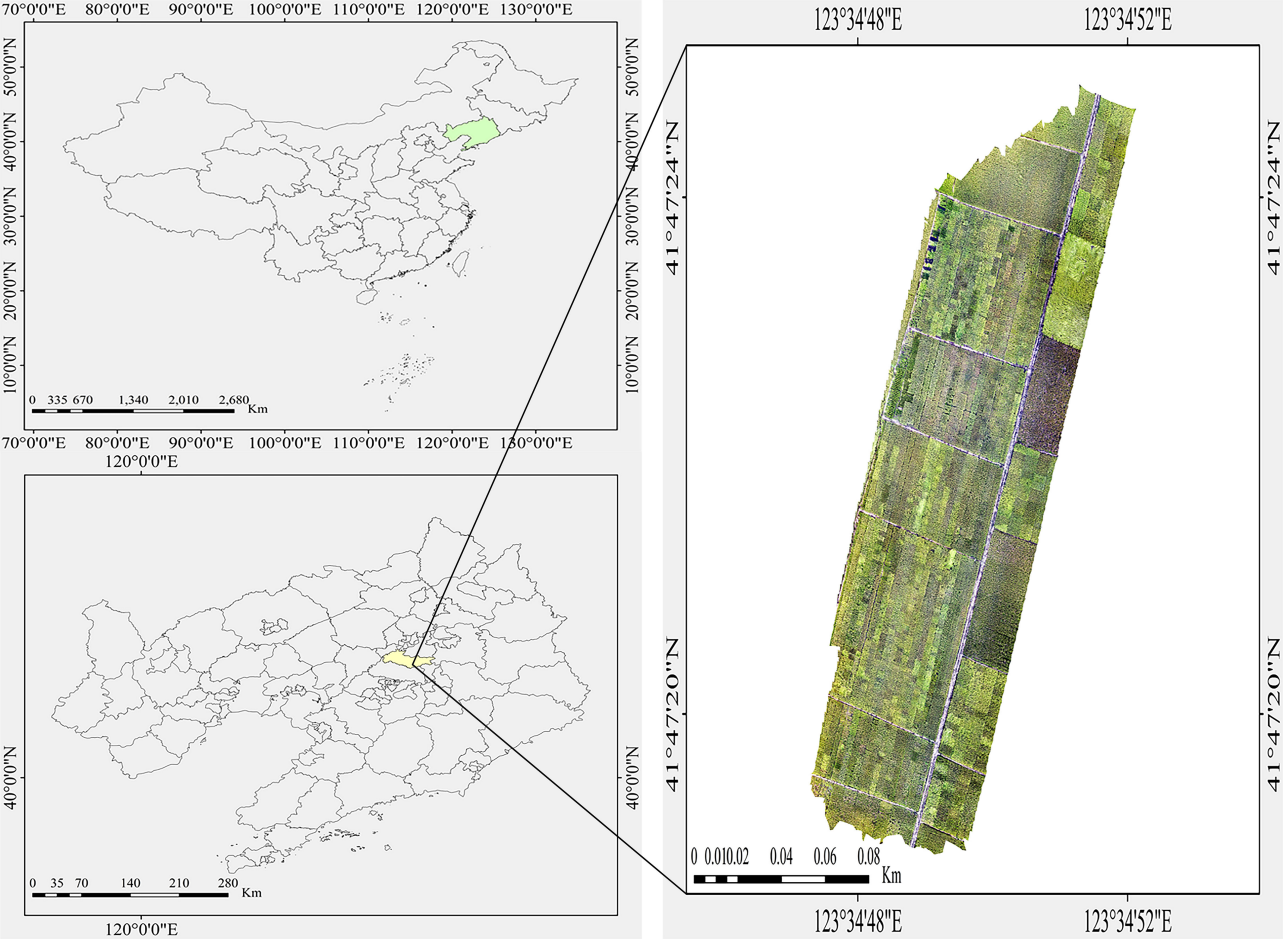
Study area.

This study conducted UAV-based field image acquisition on September 11, 2024, at the breeding experimental base of Liaoning Rice Research Institute (123.45°E, 41.80°N). This period coincided with the late grain-filling stage when panicle shapes were stable, and rice panicles were fully developed with stable morphology, ensuring that UAVs could capture clear panicle contour images. Data collection employed a DJI Mavic 3 Multispectral drone equipped with a 4/3 CMOS image sensor (20 MP effective resolution). To avoid cloud cover and ensure sufficient light, the image acquisition was carried out under clear and cloudless weather conditions, specifically from 10:00 to 14:00. The minimum flight altitude was set at 12 meters to guarantee sufficient image resolution for subsequent analysis. The UAV autonomously followed predefined flight paths with 80% front overlap and 60% side overlap settings to ensure comprehensive image coverage. Acquired nadir-view RGB images featured a resolution of 5280×3956 pixels, with camera parameters configured as follows: ISO range 100-6400, 84°field of view equivalent to 24mm focal length, aperture adjustable from f/2.8 to f/11, and focus range set from 1 meter to infinity, guaranteeing full-field scene clarity throughout the imaging area. The resulting UAV image of the study area is presented in **Figure 1**.

### 2.2 Dataset production

**Figure 2:**
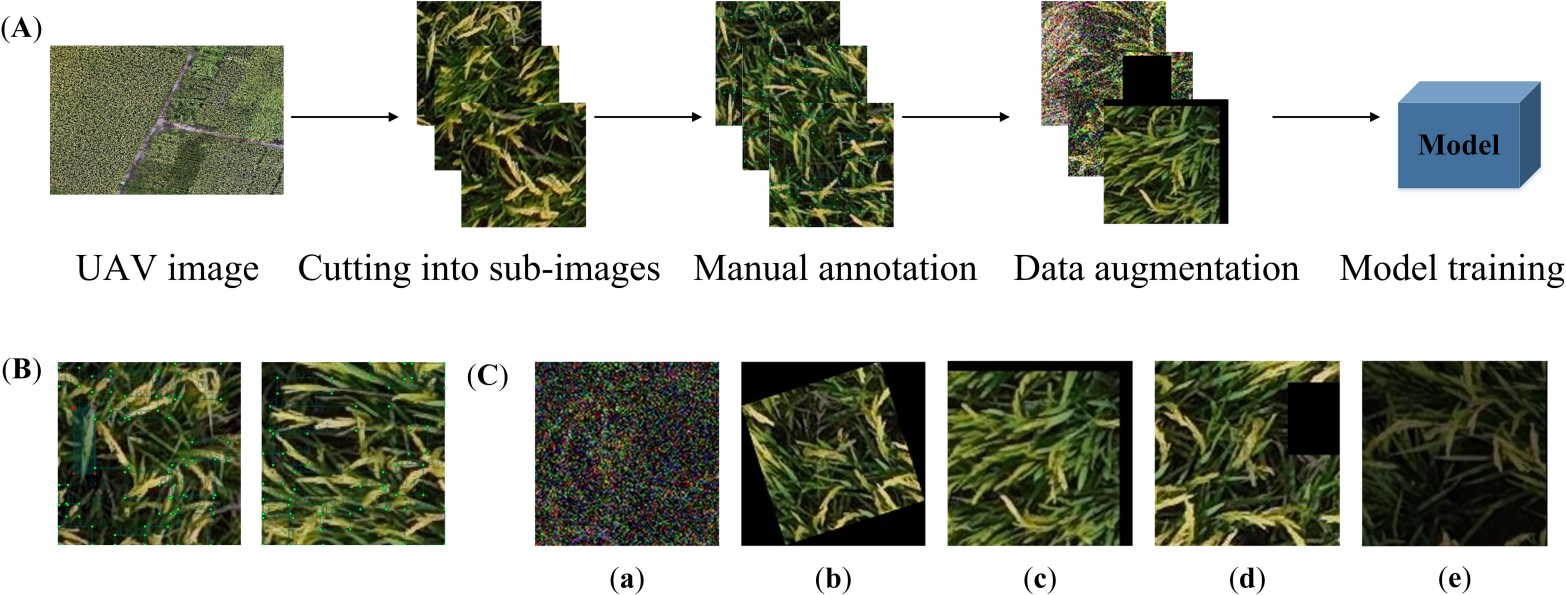
Dataset production workflow. **(A)** Image preprocessing procedure. **(B)** Annotation examples. **(C)** Data augmentation examples: (a) Noise addition. (b) Rotation. (c) Translation. (d) Random occlusion. (e) Brightness reduction.

The dataset production workflow is shown in **Figure 2**, involving key steps such as preprocessing, sample cropping, manual annotation, and data augmentation. First, preprocessing operations including grayscale stretching and geometric correction were performed on the original images. Subsequently, the preprocessed images were cropped using a sliding window with a 0.6 overlap ratio, generating 306 original samples of 128×128 pixels. The rice panicles were manually annotated using LabelImg software, and the annotation results were saved in XML file format, including object bounding box coordinates and category information.

To enhance the model’s generalization ability, data augmentation operations including noise addition, random occlusion, rotation, translation, and brightness reduction were performed on the obtained rice panicle images and annotation files. After data augmentation, the final dataset comprises 4545 images, with 4131 images in the training set and 414 images in the validation set.

### 2.3 Improved YOLOv11x model for rice panicle detection

#### 2.3.1 YOLOv11x model

As a deep learning model widely used in the field of object detection, the YOLO model has undergone multiple versions of iterative upgrades since its proposal in 2015, achieving significant improvements in detection accuracy, speed, and computational efficiency (Ye et al., 2024). Among them, YOLOv11, officially released by the YOLO community in 2024, is the latest version of the series. YOLOv11 expands and upgrades based on the architecture of YOLOv8, significantly enhancing object detection performance by achieving the best balance between speed and accuracy (Ali and Zhang, 2024).

**Figure 3:**
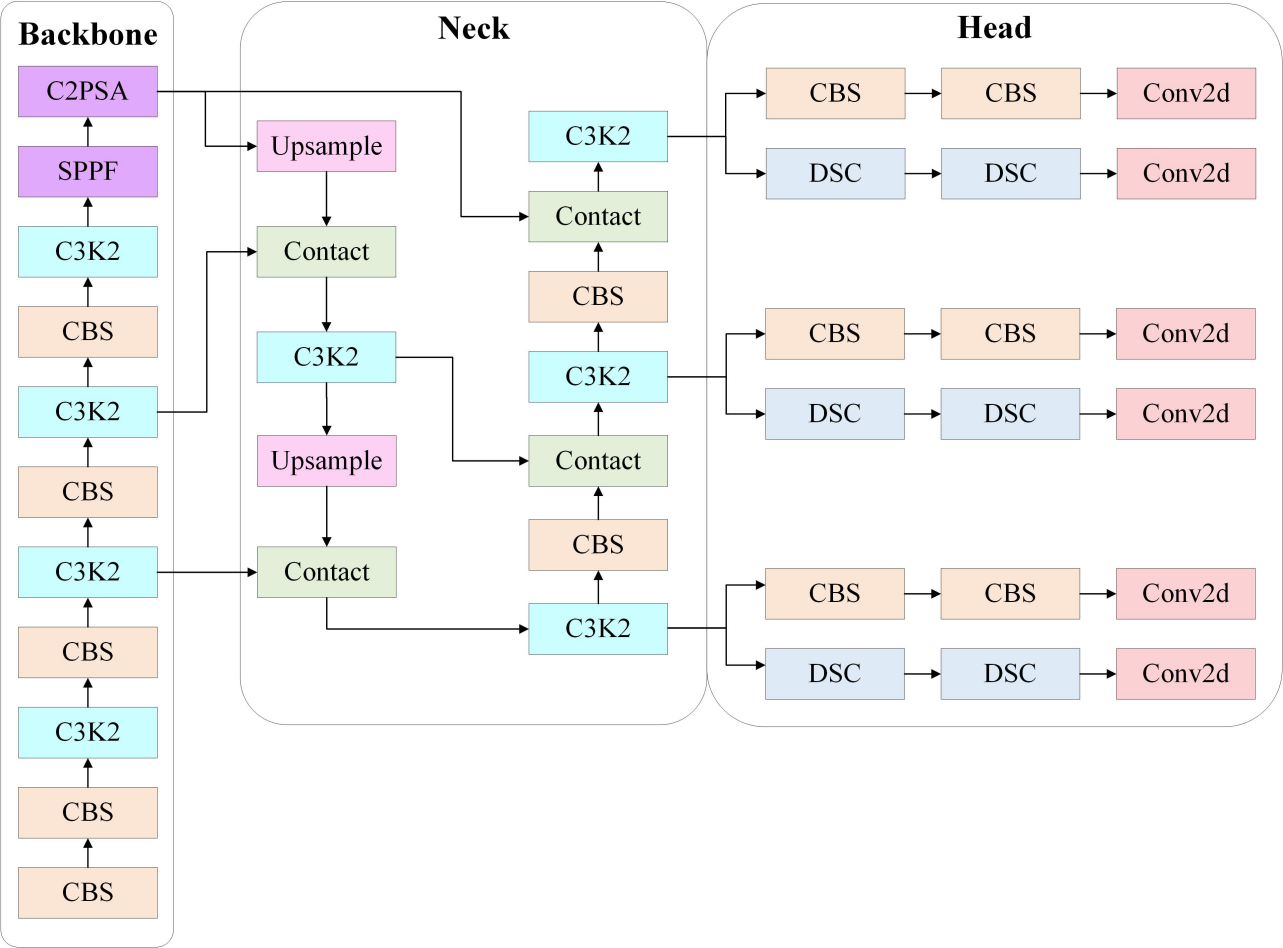
Architecture of YOLOv11.

The main architecture of YOLOv11 is shown in **Figure 3**. The backbone, a vital part of the YOLO framework, is responsible for extracting multi-scale features from the input image. YOLOv11’s backbone consists of convolutional layers, C3k2 modules, SPPF modules, and C2PSA modules. The structure of the convolutional layers is similar to previous YOLO versions, enabling feature extraction from the image. The C3k2 module, a new improvement introduced in YOLOv11, replaces the C2f module used in previous versions, contributing to enhanced computational efficiency. YOLOv11 retains the SPPF module but introduces a new C2PSA module afterward. The C2PSA module embeds a multi-head attention mechanism within a C2 module, enhancing spatial attention in feature maps and improving detection accuracy for smaller or partially occluded objects. The neck integrates features across various scales and transfers them to the head for prediction. After upsampling and concatenation, YOLOv11’s neck connects C3k2 modules in series to boost the speed and performance of the feature aggregation process. The head processes the feature maps from the neck and outputs predictions for object detection and classification. YOLOv11’s head employs two Depthwise Convolution (DWConv) layers that only handle spatial convolution, no longer processing channel-wise convolution, effectively reducing parameter computation and improving computational efficiency.

YOLOv11^1^ offers five distinct model variants: YOLOv11x, YOLOv11l, YOLOv11m, YOLOv11s, and YOLOv11n. This paper selects YOLOv11x, which demonstrates the highest detection accuracy and the best recognition performance, as the foundational framework for subsequent improvements.

#### 2.3.2 Improved YOLOv11x model

**Figure 4:**
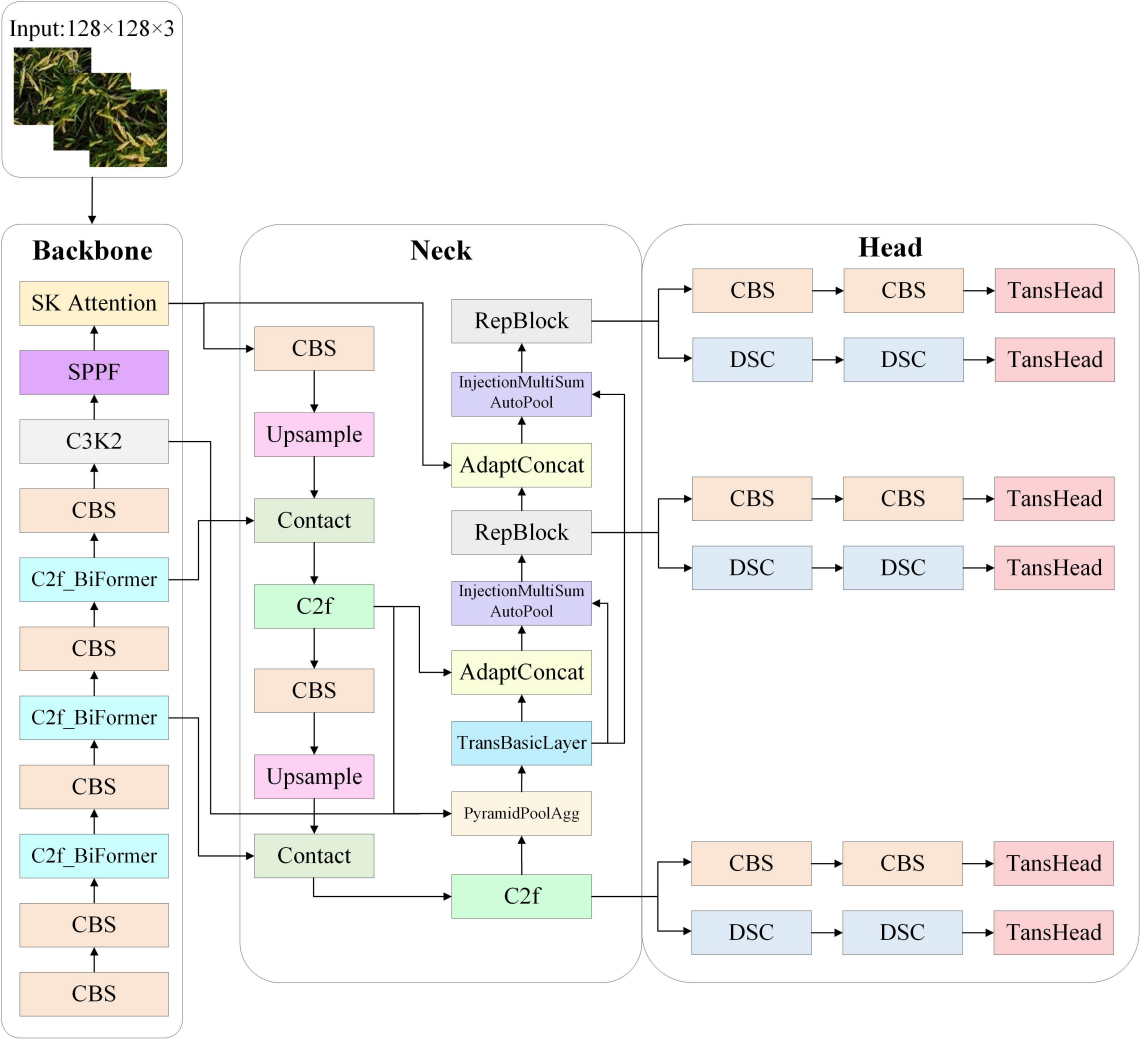
Architecture of improved YOLOv11.

This paper focuses on detecting in-field rice panicles, characterized by their high density, small size, dense distribution patterns, and frequent occlusion. Although YOLOv11x demonstrates superior detection capabilities, its accuracy in addressing these complex agricultural small-object detection challenges remains insufficient. To overcome the limitations of YOLOv11x for in-field panicle detection, we propose an improved YOLOv11x model incorporating four key architectural modifications: backbone network improvement, detection head improvement, attention mechanism improvement, and feature fusion improvement. The modified architecture is illustrated in **Figure 4**.

#### 2.3.3 Improved YOLOv11x backbone

When dealing with in-field panicle detection, the panicle images captured by UAVs are characterized by complex backgrounds, low object resolution, and dense distribution. To address the above issues, a BiFormer module based on the Bi-level Routing Attention (BRA) mechanism is introduced into the YOLOv11x backbone network.

**Figure 5:**
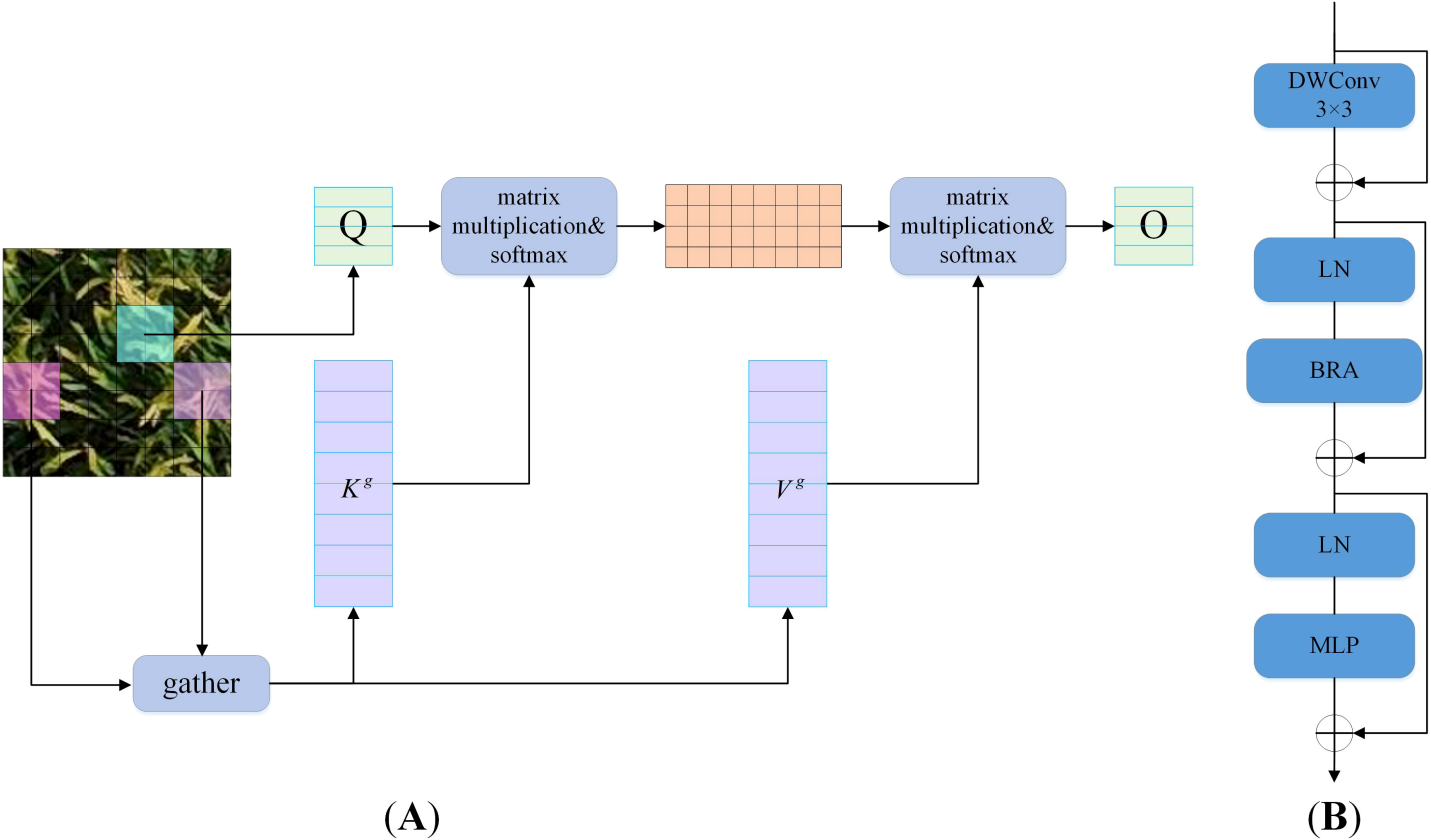
Structure of BRA and BiFormer. **(A)** BRA. **(B)** BiFormer.

Attention mechanisms constitute a core building module in vision Transformers, enabling the modeling of long-term dependencies. Traditional attention mechanisms require computing pairwise token interactions across all spatial positions, resulting in high computational complexity and substantial memory overhead. While existing research has proposed improvements such as local windows, axial stripes, dilated windows, and deformable attention, most methods aim to address this challenge by introducing handcrafted, content-agnostic sparsity into attention computation. BRA that proposed by Zhu et al. (2023) addresses scalability challenges in Multi-Head Self-Attention (MHSA) through a dynamic, query-aware sparse attention mechanism. Its workflow, illustrated in **Figure 5A**, operates as follows: For a query, most irrelevant key-value pairs are first filtered out at the coarse-grained region level, and then fine-grained token-to-token attention is applied exclusively within the union of remaining candidate regions.

BiFormer (Zhu et al., 2023) is a general-purpose vision Transformer that uses BRA as its fundamental building module. It follows the architectural design of most vision Transformers with a four-stage pyramid structure. Specifically, BiFormer comprises four sequential modules. Each module first applies overlapping patch embedding, then increases the number of channels and reduces the input spatial resolution through patch merging, and finally performs feature transformation via stacked BiFormer modules. **Figure 5B** illustrates the structural diagram of the BiFormer module, comprising four key components: DWConv, Layer Normalization (LN), BRA, and Multi-Layer Perceptron (MLP). Among them, the DWConv is used to implicitly encode relative position information, reducing model parameters and computational complexity. The LN accelerates training and improves the model’s generalization ability. The MLP adjusts the attention weights to enhance focus on different features. By employing the BRA mechanism, the BiFormer module can focus on important features at different levels, thereby paying more attention to regions containing small objects and extracting more accurate features. Meanwhile, BiFormer uses a pyramid network structure to achieve multi-scale object detection. Additionally, sparse sampling in BiFormer better preserves the fine-grained features of small objects from UAV perspectives and enables more precise selection of receptive fields.

#### 2.3.4 Improved YOLOv11x head

This paper applies a Transformer-based detection head (TransHead) into YOLOv11 to improve the model’s predictive performance for small objects (Zhu et al., 2021). The core of TransHead lies in leveraging the powerful ability of Transformers to model long-term dependencies. In object detection tasks, traditional detection heads are often built based on convolutional neural networks (CNNs). Convolutional operations mainly concentrate on local information and have limited capabilities in capturing long-term dependencies between objects and global contextual information. By replacing the original detection head of YOLOv11 with TransHead, the model can adaptively focus on features at different positions in the image through the self-attention mechanism of Transformers. This enables the model to better capture associations between objects and global contextual information, thus enhancing its adaptability to complex scenarios and performance in detecting small objects.

#### 2.3.5 SK Attention

**Figure 6:**
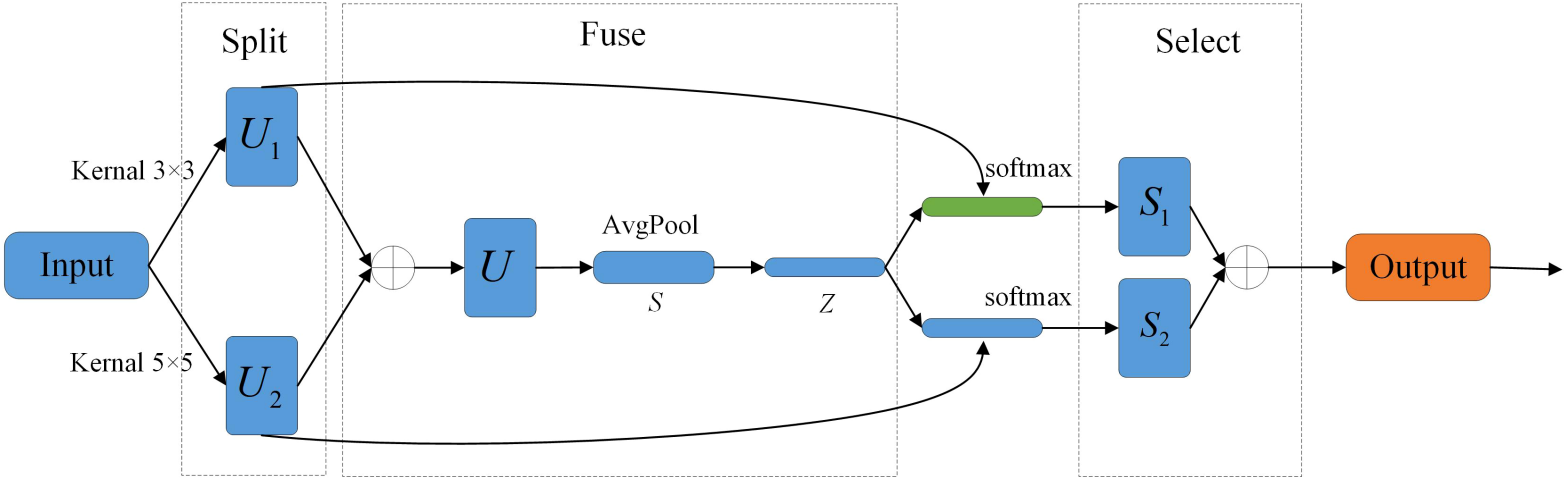
Structure of SK Attention.

The structure of the Selective Kernel (SK) Attention mechanism (Li et al., 2019) is illustrated in **Figure 6**, comprising three operations: Split, Fuse, and Select. The Split operation employs 3×3 and 5×5 convolutional kernels to process input images, generating two feature maps denoted *U*_1_ and *U*_2_. During the Fuse stage, weights for both kernels are computed, and the feature maps *U*_1_ and *U*_2_ undergo element-wise summation to produce feature map *U*. Global average pooling is then applied to *U*, yielding feature vector *S*. This vector undergoes dimensionality reduction via a fully connected layer, producing reduced-dimension vector *Z*. Two additional fully connected layers subsequently restore dimensionality, generating feature vectors matching the original dimensions. The Select operation employs the softmax activation function for normalization to compute weight scores corresponding to each channel. These weights are then applied to the feature maps, followed by information fusion of the two recalibrated feature maps to generate the final output image. Compared to the input image, the output dynamically integrates multi-scale features through selective fusion, significantly enhancing the representation of critical visual information.

#### 2.3.6 Feature fusion

In object detection models, feature fusion is one of the key techniques for improving model performance (Lian et al., 2024; Deng et al., 2020). The original YOLOv11x employs a relatively simple feature fusion strategy, which exhibits limitations when dealing with complex small object detection tasks. Therefore, this paper enhances the original YOLOv11x by incorporating multiple feature fusion modules across different scales to improve the model’s adaptability to multi-scale objects.

The improvements to the YOLOv11 feature fusion stage primarily encompass five components: RepBlock, InjectionMultiSumAutoPool, AdaptConcat, TransBasicLayer, and PyramidPoolAgg. The PyramidPoolAgg module processes input feature maps of varying sizes, endowing the model with scale invariance. By employing pooling operations at different scales to capture multi-scale image information, it effectively expands the receptive field and enhances the utilization of global information. The TransBasicLayer module integrates a Transformer mechanism, which has demonstrated exceptional feature extraction capabilities across multiple fields like natural language processing and object detection. Leveraging this mechanism, it extracts higher-level features from the output of the PyramidPoolAgg module, providing high-quality input feature maps for subsequent network operations. The AdaptConcat module performs adaptive average pooling on two input feature layers of different sizes before concatenation. The InjectionMultiSumAutoPool module accounts for the disparities between nearby local feature layers and distant global feature layers. By assigning weights to local feature layers and combining them with global layers, it creates a new feature layer that significantly highlights objects during training, improving the model’s ability to capture and recognize key information. The RepBlock module operates on structural reparameterization technology, which enables the model to adopt a multi-branch structure during training to learn richer features and enhance expressiveness, while transforming into a single-branch structure during inference to reduce computational overhead and improve efficiency and speed.

#### 2.3.7 Model training

The rice panicle detection model presented in this paper was trained and tested on a Windows 10 (64-bit) operating system. The computing environment comprised an INTEL(R) XEON(R) GOLD 6530 CPU, an NVIDIA L40S GPU, and 881 GB RAM. The experiments were conducted using PyTorch 2.0.1 as the deep learning framework, with CUDA 11.8 and CUDNN 8.7.0.0 for GPU acceleration. Python served as the programming language. Prior to training, the network was initialized with weights pre-trained on the YOLO dataset. Hyperparameter configurations for the model training phase are detailed in **Table 1**.

**Table 1:** Hyperparameter configurations.

Hyperparameters	Configuration
Optimizer	SGD
Momentum	0.9
Initial learning rate	0.001
Weight decay	0.0005
Epoch	200

#### 2.3.8 Performance metrics

This paper employs Precision (P), Recall (R), F1-score, and mean Average Precision (mAP) as performance metrics to evaluate the detection effect of the rice panicle detection model under complex field conditions. The expressions of these four metrics are shown in Equations 1–5:


(1) $$\text{Precision}=\frac{TP}{TP+FP}\times 100\%$$

(2) $$\text{Recall}=\frac{TP}{TP+FN}\times 100\%$$

(3) $$\text{F}1-\text{score}=2\times \frac{Precision\times Recall}{Precision+Recall}$$

(4) $$\text{mAP}=\frac{1}{C}\sum_{i=1}^{C}AP_{i}\times 100\%$$

where


(5) $$AP=\int_{0}^{1}P(R)dR$$

*TP*, *TN*, *FP*, and *FN* denote the numbers of true positives, true negatives, false positives, and false negatives, respectively, and *C* denotes the number of classes.

Precision refers to the proportion of true positives out of all the positive predictions made by the model. Recall is defined as the proportion of true positives out of all the actual positives. These two metrics reflect the accuracy and coverage of the model’s predictions. The F1-score, calculated as the harmonic mean of Precision and Recall, serves as a comprehensive evaluation metric that balances both measures to avoid overemphasis on a single extreme value, thus providing an overall evaluation of model performance. The mAP comprehensively evaluates the model’s detection accuracy across different classes (Zhang et al., 2024). It is calculated by computing the Average Precision (AP) for each class and then taking the mean of these values. In this paper, mAP with an Intersection over Union (IoU) threshold of 0.5 (mAP@0.5) is used to measure the model’s detection performance, where a higher mAP@0.5 value indicates better detection capabilities.

In addition, for the rice panicle counting results, this paper uses the coefficient of determination (R^2^), root mean square error (RMSE), and relative RMSE (rRMSE) as performance metrics to evaluate the accuracy of the model in counting rice panicles.Their expressions are shown in Equations 6–8:


(6) $${\text{R}}^{2}=1-\frac{\sum_{i=1}^{n}{\left(y_{i}-{\hat{y}}_{i}\right)}^{2}}{\sum_{i=1}^{n}{\left(y_{i}-{\bar{y}}_{i}\right)}^{2}}\;$$

(7) $$\text{RMSE}=\sqrt{\frac{1}{n}\sum_{i=1}^{n}{\left(y_{i}-{\hat{y}}_{i}\right)}^{2}}$$

(8) $$\text{rRMSE}=\frac{\sqrt{\frac{1}{n}\sum_{i=1}^{n}{\left(y_{i}-{\hat{y}}_{i}\right)}^{2}}}{{\bar{y}}_{i}}$$

where *y_i_*, ${\hat{y}}_{i}$ and ${\bar{y}}_{i}$ represent the true value, predicted value, and mean true value of rice panicle counting in the test samples, respectively.

## 3 Results

### 3.1 Ablation experiments

To verify the effectiveness of the improved method proposed in this paper, ablation experiments are carried out on the same dataset to determine the effect of each improvement point. Based on the original YOLOv11x model, improvements such as backbone, head, SK Attention, and feature fusion are sequentially added, with the remaining model training parameters kept the same. The results of the ablation experiments are shown in **Table 2**, where “✓” and “×” indicate the use and non-use of the corresponding improvements, respectively.

**Table 2:** Results of ablation experiments.

Backbone	Head	SK Attention	Feature fusion	Precision	Recall	F1-score	mAP@0.5
×	×	×	×	84.3%	78.6%	81.4%	86.4%
✓	×	×	×	84.4%	80.0%	82.1%	87.5%
✓	✓	×	×	85.2%	80.1%	82.6%	87.7%
✓	✓	✓	×	86.9%	80.4%	83.5%	88.5%
✓	✓	✓	✓	87.3%	81.0%	84.1%	89.4%

From **Table 2**, it can be seen that after introducing the backbone improvement, the Recall of the model increases by 1.4%, and mAP@0.5 increases by 1.1%. After introducing the backbone and head improvements, all performance metrics of the model are significantly improved, with the F1-score increasing by 1.2% and mAP@0.5 increasing by 1.3%. Further adding SK Attention to the model improves the Precision by 2.6% and mAP@0.5 by 2.1%. Finally, by improving the network structure of the feature fusion stage, the model achieves an mAP@0.5 of 89.1%, an increase of 2.7% compared to the original network.

The ablation results above clearly demonstrate the positive contributions of each improved module to the model’s performance, while the synergistic effects of these modules further validate the rationality of the overall architecture design. BRA dynamically filters irrelevant regions through its Bi-level Routing Attention mechanism, significantly enhancing feature extraction for small objects in densely occluded environments. TransHead leverages the global modeling capability of Transformers to compensate for the limitations of traditional CNN heads in capturing long-term spatial dependencies. SK Attention improves the model’s adaptability to scale variations through dynamic multi-scale kernel selection, while the multilevel feature fusion module further integrates local details with global context via cross-scale information interaction. Experimental results demonstrate that the synergistic effects of these four components lead to a significant improvement in detection accuracy for in-field rice panicle detection using UAV images.

### 3.2 Comparison with other detection models

#### 3.2.1 Comparison of detection results

The improved rice panicle detection model based on YOLOv11x is compared with current mainstream object detection algorithms. All detection models use the same dataset and data augmentation methods, and the model training parameters are all identical. The performance metrics of the five detection models are presented in **Table 3**.

**Table 3:** Comparison of performance metrics of different models.

Model	Precision	Recall	F1-score	mAP@0.5
Faster R-CNN	76.4%	90.0%	82.6%	77.0%
YOLOv8	81.1%	77.9%	79.5%	85.7%
YOLOv11l	81.5%	79.1%	80.3%	86.3%
YOLOv11x	84.3%	78.6%	81.4%	86.4%
Improved YOLOv11x (Our method)	87.3%	81.0%	84.1%	89.4%

As presented in **Table 3**, the method proposed in this paper achieves the highest Precision (87.3%), F1score (84.1%) and mAP@0.5 (89.4%). Compared with the baseline model YOLOv11x, the improved model increases Precision by 3%, Recall by 2.4%, F1-score by 2.7% and mAP@0.5 by 3%. When compared with the widely used YOLOv8 model, the improved model demonstrates a 6.2% increase in Precision, 3.1% in Recall, 4.6% in F1-score and 3.7% in mAP@0.5. In comparison with the YOLOv11l model, the improved model shows a 5.8% improvement in Precision, 1.9% in Recall, 3.8% in F1-score and 3.1% in mAP@0.5. Although the improved model has a slight decrease in Recall when compared with the Faster R-CNN model, it achieves a 1.5% increase in F1-score and a significant 12.4% increase in mAP@0.5.

These results clearly demonstrate that the improved model proposed in this paper possesses higher detection accuracy and exhibits superior detection performance. It outperforms both the baseline model and other mainstream object detection algorithms in multiple key performance metrics, which benefits from the synergistic effect of the introduced BRA, TransHead, SK Attention mechanism, and optimized feature fusion module. These components collectively enhance the model’s ability to capture densely distributed, large scale-varied, and occluded rice panicles in UAV images, validating its effectiveness and advancement in in-field rice panicle detection tasks.

**Figure 7:**
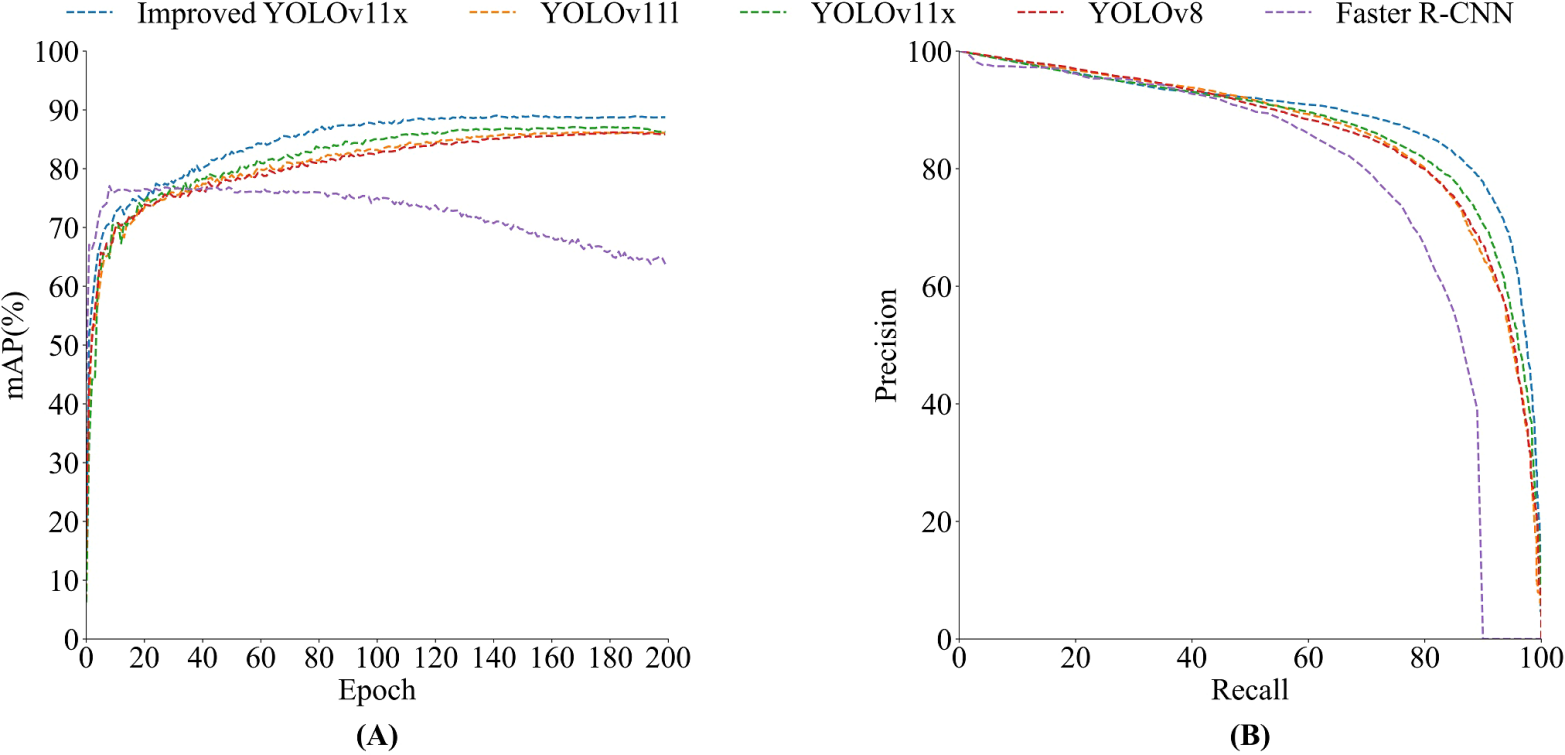
The mAP curves and P-R curves of different network models. **(A)** mAP curves. **(B)** P-R curves.

**Figure 8:**
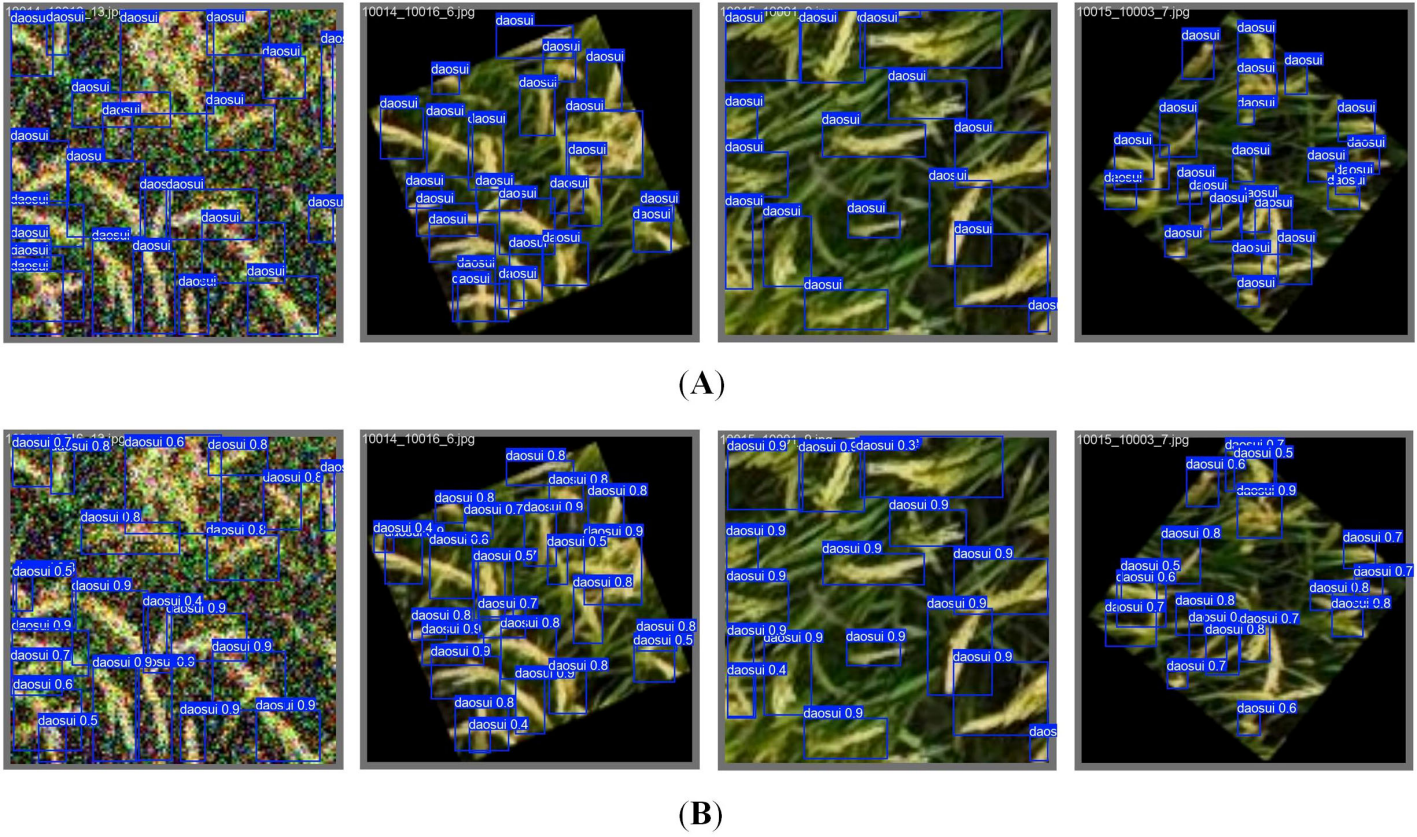
Recognition results of the improved model. **(A)** Annotated images. **(B)** Recognition results of the improved YOLOv11x model.

**Figure 7** shows the mAP curves and P-R curves of different network models. As depicted in **Figure 7A**, the mAP values of different models eventually converges gently as the number of iterations increases, and the improved model exhibits a higher convergence accuracy. **Figure 7B** reveals that the area under the P-R curve of the improved model is closer to 1, indicating that the algorithm’s detection performance is superior. Additionally, as can be seen from **Figure 8**, the improved model is capable of covering the rice panicle regions with highly accurate bounding boxes for manually annotated panicles. Even when faced with variations in panicle morphology and background interference, the model can still effectively detect the objects, demonstrating its robustness in the task of rice panicle detection in UAV images under field conditions.

**Figure 9:**
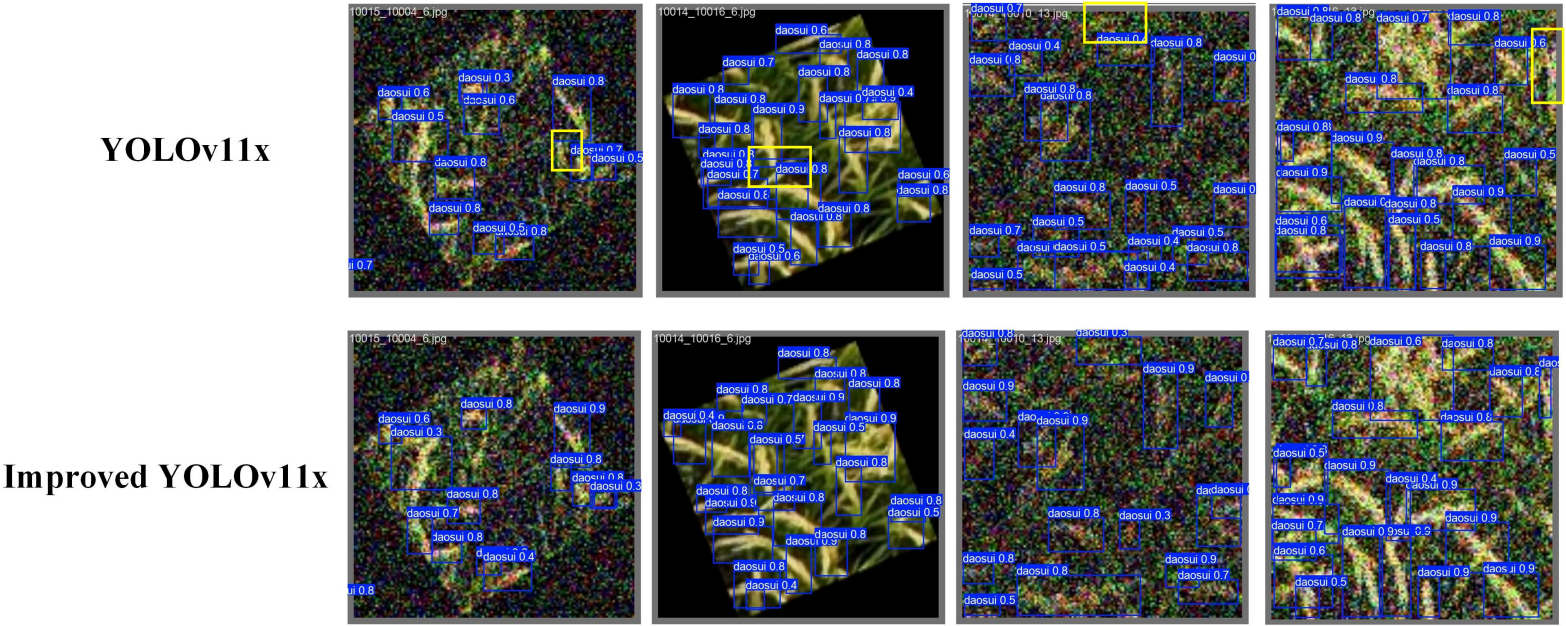
Comparison of recognition results between our proposed method and the original YOLOv11x model (Yellow boxes indicate missed detections).

The comparison of recognition results between our proposed method and the original YOLOv11x model is shown in **Figure 9**. It can be seen that there are missed detections in the recognition results of the original YOLOv11x model. In contrast, our method optimizes the network structure based on YOLOv11x, which effectively eliminates such missed detection cases. Consequently, the improved model demonstrates significantly enhanced capability in detecting small objects in complex field scenarios, thereby exhibiting superior performance in-field rice panicle detection.

#### 3.2.2 Comparison of rice panicle counting results

**Figure 10:**
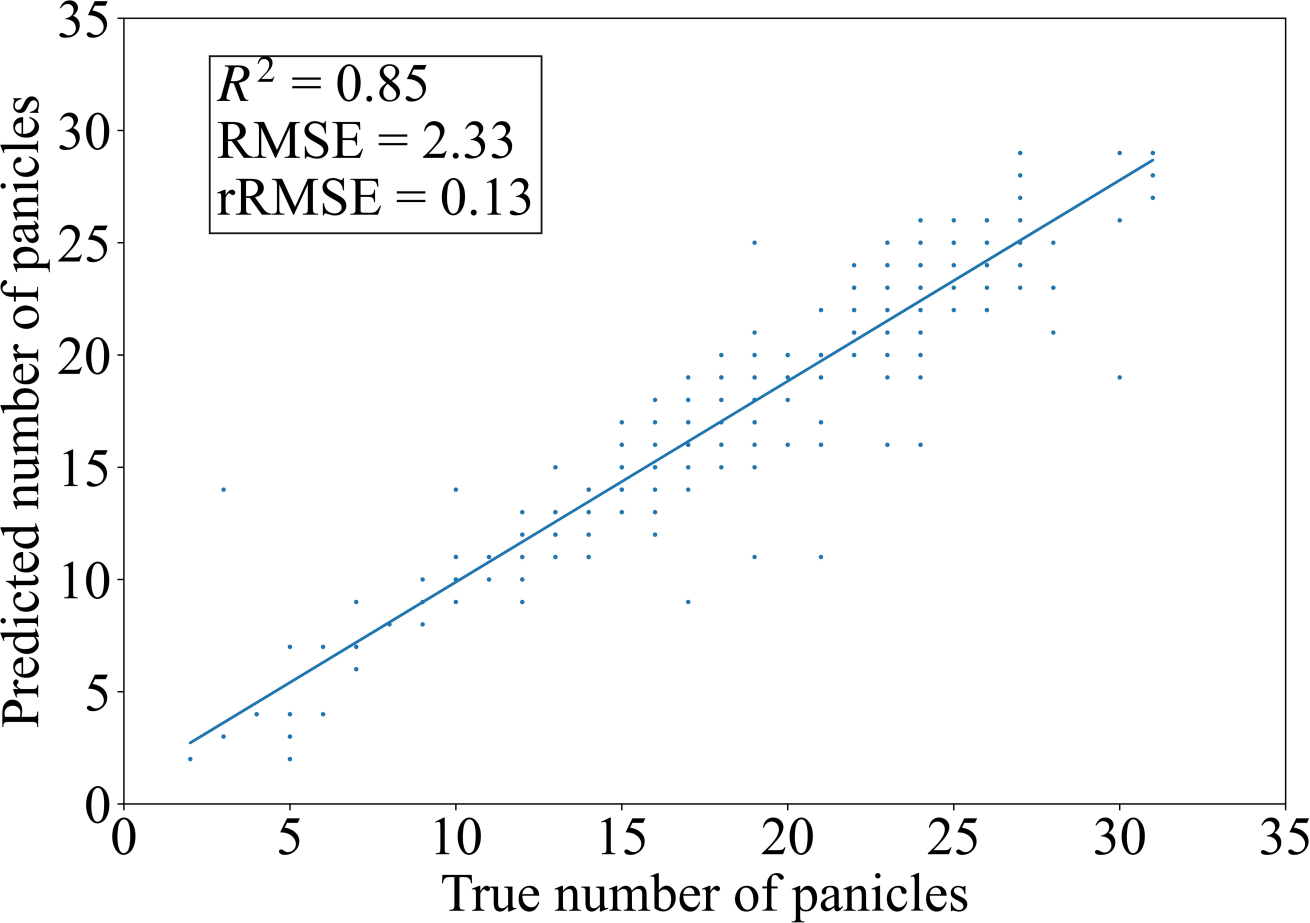
Comparison between the true number of rice panicles and the number predicted by the improved YOLOv11x model.

To verify the counting accuracy of the rice panicle detection model, 300 rice panicle images are selected for testing, and Non-Maximum Suppression (NMS) is used to achieve rice panicle counting. The confidence threshold is set as p=0.5 and the IoU threshold is set as IoU=0.5. The counting metrics of the proposed rice panicle detection model and the other four compared models are shown in **Table 4**. It can be seen that the counting results of our method are R^2^ = 0.85, RMSE=2.33, and rRMSE=0.13, which are all the best among the five algorithms. Meanwhile, from the comparison between the true number of rice panicles and the number predicted by our model shown in **Figure 10**, it can be seen that the predicted values can well fit the true values, further verifying the counting accuracy of the proposed model. The counting results of the other compared models are shown in **Supplementary Figures S1–4**.

**Table 4:** Comparison of performance metrics for rice panicle counting results.

Model	R2	RMSE	rRMSE
Faster R-CNN	0.54	4.12	0.22
YOLOv8	0.60	3.84	0.21
YOLOv11l	0.66	3.57	0.19
YOLOv11x	0.82	2.58	0.14
Improved YOLOv11x (Our method)	0.85	2.33	0.13

## 4 Discussion

Experimental results in Section 3 demonstrate that the enhanced performance of our proposed improved YOLOv11x model for in-field rice panicle detection and counting arises from the synergistic interplay of its various modified modules. Specifically, the BRA mechanism integrated into the backbone network operates via a dynamic feature screening process: it first identifies potential rice panicle regions at the coarse-grained level, followed by fine-grained token-to-token attention computations within the retained candidate regions. This dual-step approach effectively mitigates interference from complex background noise and strengthens the model’s capability to extract features of high-density, small-sized panicles.

The original detection head is replaced with the TransHead, which leverages the self-attention mechanism inherent to Transformers. This modification enhances the model’s capacity to capture long-term dependencies, allowing it to better associate the morphological and structural features of rice panicles with contextual information. Consequently, detection accuracy is improved in scenarios characterized by mutual occlusion and complex backgrounds.

The incorporation of the SK Attention mechanism enables the model to dynamically select and fuse multi-scale features, thereby strengthening the perception of key panicle information. Additionally, the multi-scale feature fusion module further integrates local details with global context, effectively boosting the model’s adaptability to rice panicles of varying growth states and scales in field environments. Together, the synergistic effect of these mechanisms drives the significant improvements in the model’s detection accuracy and counting precision.

**Table 5** demonstrates the influence of different UAV flight altitudes and input image sizes on the performance of the improved YOLOv11x model. As shown in the table, the model achieves the best performance at a UAV flight altitude of 12 meters with an input size of 128×128 pixels. Specifically, it reaches a Precision of 95.4%, an F1-score of 87.4%, and an mAP@0.5 of 84.6%. Meanwhile, the inference time for a single image is the shortest, at 0.0438 seconds. As the input size increases, the model performance shows a downward trend. When the input size is 384×384 pixels, the mAP@0.5 drops to 74.7%, and the inference time increases to 0.1189 seconds. This indicates that small-sized inputs are more conducive for the model to capture the local features of rice panicles. In contrast, large-sized inputs may introduce more background noise. This makes feature extraction more difficult and leads to a decrease in detection accuracy and speed.

**Table 5:** Performance metrics of the improved YOLOv11x with different input image sizes at various UAV flight altitudes.

UAV flight altitude	Input image size	Precision	Recall	F1-score	mAP@0.5	Time (s)
12 m	128×128 256×126	95.4% 77.1%	80.6% 76.6%	87.4% 76.9%	84.6% 78.1%	0.0438 0.0871
	300×300	81.0%	71.3%	75.8%	76.7%	0.1140
	384×384	79.2%	73.7%	76.4%	74.7%	0.1189
15 m	128×128 256×126	89.4% 74.4%	61.1% 65.0%	72.6% 69.4%	73.8% 68.9%	0.0701 0.1151
	300×300	67.8%	58.8%	63.0%	63.4%	0.1175
	384×384	60.5%	55.7%	58.0%	58.6%	0.1213

When the UAV flight altitude increases to 15 meters, the model performance generally declines. For example, with an input size of 128×128 pixels, the Precision drops to 89.4%, the mAP@0.5 decreases to 73.8%, and the inference time increases to 0.0701 seconds. This phenomenon indicates that the increase in flight altitude reduces image resolution and causes the loss of object detail information, thereby affecting the model’s detection ability for small objects. Additionally, when the input size is 384×384 pixels, the model performance further deteriorates. The mAP@0.5 is 58.6%, and the inference time is 0.1213 seconds. This suggests that under high UAV flight altitudes, large-sized inputs exacerbate the problem of object blurring and further weaken the model performance.

It is worth noting that the improved YOLOv11x-based rice panicle detection model proposed in this paper exhibits significant differences in model architecture and applicable scenarios compared with existing studies (Seng et al., 2025; Liang et al., 2024). In terms of model architecture, the latter two are both based on YOLOv5, while this paper is built on the more advanced YOLOv11x, whose native architecture has better feature extraction efficiency and parameter balance, providing a stronger foundation for improvements. Regarding applicable scenarios, Seng et al. (2025) focus on lightweight detection for near-ground high resolution images; although Liang et al. (2024) realize in-field rice panicle detection using UAVs, their work targets low-altitude scenarios with a flight altitude of only 1.1–3 meters. In contrast, this paper is aimed at UAV images captured at 12–15 meters. In complex scenarios with dense panicles and large scale variations, it achieves an mAP@0.5 of 89.4% and counting results with R^2^ = 0.85 and RMSE=2.33, making it more suitable for large-scale field monitoring.

## 5 Conclusion

This paper proposes an improved rice panicle detection model based on YOLOv11x for detecting rice panicles in UAV images with complex field backgrounds. In the improved YOLOv11x model, the BiFormer module is introduced into the backbone network to enhance the feature extraction capability for small objects. The Transformer-based detection head is adopted to capture long-term dependency relationships. The SK Attention mechanism is combined to dynamically fuse multi-scale features, and the feature fusion module is optimized to improve multi-scale adaptability. Ablation experiments verify the effectiveness of each improved module. Comparative experiments show that the improved model achieves a Precision of 87.3%, an F1-score of 84.1%, and an mAP@0.5 of 89.4%, significantly outperforming the original YOLOv11x, YOLOv11l, and mainstream algorithms like YOLOv8 and Faster R-CNN. This provides practical technical support for large-scale rice panicle detection by UAVs. Panicle counting tests on 300 rice panicle images demonstrate the model’s good fitting performance, with R^2^ = 0.85, RMSE=2.32, and rRMSE=0.13. However, the detection accuracy of our model decreases when dealing with large-sized input images. In the future, we will further explore high-precision rice panicle detection technologies for large-sized UAV images of field rice.

## Data Availability

The raw data supporting the conclusions of this article will be made available by the authors, without undue reservation.

## Footnotes

1
https://github.com/ultralytics/ultralytics.

## Supplementary material

Supplementary Figure S1 Comparison between the true number of rice panicles and the number predicted by the Faster R-CNN model.

Supplementary Figure S2 Comparison between the true number of rice panicles and the number predicted by the YOLOv8 model.

Supplementary Figure S3 Comparison between the true number of rice panicles and the number predicted by the YOLOv11l model.

Supplementary Figure S4 Comparison between the true number of rice panicles and the number predicted by the YOLOv11x model.
